# Prothrombotic effects of tumor necrosis factor alpha in vivo are amplified by the absence of TNF-alpha receptor subtype 1 and require TNF-alpha receptor subtype 2

**DOI:** 10.1186/ar4064

**Published:** 2012-10-18

**Authors:** Joachim Pircher, Monika Merkle, Markus Wörnle, Andrea Ribeiro, Thomas Czermak, Yvonn Stampnik, Hanna Mannell, Markus Niemeyer, Volker Vielhauer, Florian Krötz

**Affiliations:** 1 Medizinische Klinik und Poliklinik IV, Innenstadt, Ludwig Maximilians University München, Ziemssenstr. 1, 80336 Munich, Germany; 2 Walter Brendel Centre of Experimental Medicine, Ludwig Maximilians University München, Marchioninistr. 27, 81377 Munich, Germany; 3 Department of Gynecology, Klinikum rechts der Isar, Technische Universität München, Ismaninger Strasse 22, Munich, Germany; 4 Invasive Cardiology, Clinic Starnberg, Oßwaldstr. 1, 82319 Munich, Germany

## Abstract

**Introduction:**

Elevated serum levels of the proinflammatory cytokine tumor necrosis factor alpha (TNFα) correlate with an increased risk for atherothrombotic events and TNFα is known to induce prothrombotic molecules in endothelial cells. Based on the preexisting evidence for the impact of TNFα in the pathogenesis of autoimmune disorders and their known association with an acquired hypercoagulability, we investigated the effects of TNFα and the role of the TNF receptor subtypes TNFR1 and TNFR2 for arteriolar thrombosis in vivo.

**Methods:**

Arteriolar thrombosis and platelet-rolling in vivo were investigated in wildtype, TNFR1-/-, TNFR2-/- and TNFR1-/R2-/- C57BL/6 mice using intravital microscopy in the dorsal skinfold chamber microcirculation model. In vitro, expression of prothrombotic molecules was assessed in human endothelial cells by real-time PCR and flow cytometry.

**Results:**

In wildtype mice, stimulation with TNFα significantly accelerated thrombotic vessel occlusion in vivo upon ferric chloride injury. Arteriolar thrombosis was much more pronounced in TNFR1-/- animals, where TNFα additionally led to increased platelet-endothelium-interaction. TNFα dependent prothrombotic effects were not observed in TNFR2-/- and TNFR1-/R2- mice. In vitro, stimulation of human platelet rich plasma with TNFα did not influence aggregation properties. In human endothelial cells, TNFα induced superoxide production, p-selectin, tissue factor and PAI-1, and suppressed thrombomodulin, resulting in an accelerated endothelial dependent blood clotting in vitro. Additionally, TNFα caused the release of soluble mediators by endothelial cells which induced prothrombotic and suppressed anticoagulant genes comparable to direct TNFα effects.

**Conclusions:**

TNFα accelerates thrombus formation in an in vivo model of arteriolar thrombosis. Its prothrombotic effects in vivo require TNFR2 and are partly compensated by TNFR1. In vitro studies indicate endothelial mechanisms to be responsible for prothrombotic TNFα effects. Our results support a more selective therapeutic approach in anticytokine therapy favouring TNFR2 specific antagonists.

## Introduction

Tumor necrosis factor alpha (TNFα) is known to exert pleiotropic effects on the host defense which fundamentally differ depending on an acute or chronic release, the occurrence of a disequilibrium between pro- and anti-inflammatory mediators, and the concomitant regulation of other cytokines sharing certain biological properties. Trimeric TNFα acts by binding to one of the TNFα receptors (TNFRs): TNFR1 or TNFR2 [1,2]. Whereas TNFR1 is widely expressed, expression of TNFR2 is found mainly on immune and endothelial cells [3,4]. The receptors have different affinity for the soluble and the membrane-bound forms of TNFα (transmembrane TNFα, or tmTNFα); TNFR1 binds the two forms equally well, whereas TNFR2 has a higher affinity for tmTNFα. Both receptor subtypes are associated with the activation of nuclear factor-kappa-B (NF-κB). However, the signaling pathways are complex and partly crosslinked, and TNFα is able to induce cell death as well as proliferation, differentiation, and migration [5].

The impact of TNFα in the pathogenesis of autoimmune disorders such as systemic lupus erythematosus and rheumatoid arthritis (RA) has been widely accepted [6]. TNFα is known to mediate synovial cell proliferation and bone resorption in RA [7] as well as activation of endothelial cells and fibroblast chemotaxis in systemic sclerosis [8]. In patients with systemic lupus erythematosus, high serum levels of TNFα correlating with disease activity have been described, and its role in the development of lupus nephritis is accepted [9]. Additionally, patients affected by RA are at increased risk for cardiovascular morbidity and atherothrombotic events not ascribable to traditional risk factors [10-12]. Not only is atherosclerosis considered to be an inflammatory process with increased serum levels of TNFα present in patients with atherothrombotic diseases [13,14], but TNFα has been associated directly to endothelial dysfunction by mediating NF-κB translocation, increasing production of reactive oxygen species, and affecting endothelial nitric oxide synthase expression [15-17]. In addition, systemic inflammation implicates prothrombotic conditions by upregulation of procoagulant factors, downregulation of natural anticoagulants, and inhibition of fibrinolysis. More specifically, TNFα induces the expression of tissue factor and suppresses thrombomodulin as well as the endothelial cell protein C receptor [18,19]. Hence, an acquired hypercoagulability is frequently associated with autoimmune disorders. Finally, platelets represent a further important link between inflammation, atherogenesis, and thrombosis [20,21].

An effective blockade of TNFα-dependent cytokine cascades and leukocyte recruitment as well as good clinical and serological response rates have led to the establishment of therapy with TNFα inhibitors in RA and other autoimmune disorders. Reduction of atherosclerotic complications relating to attenuation of the acute-phase response, including C-reactive protein, platelet count, and fibrinogen, has been reported [22]. In light of the occurrence of serious side effects, though, the use of a non-selective blockade of all TNFα effects has been questioned.

In this study, we used the dorsal skinfold chamber microcirculatory model for *in vivo *assessment of arteriolar thrombosis and platelet-vessel wall interaction (PVWI) to investigate whether TNFα increases arteriolar thrombus formation *in vivo*, define the underlying platelet- or endothelium-mediated mechanisms, and specify TNFα receptor subtype-specific signaling.

## Materials and methods

### Chemicals

Murine TNFα was from Chemicon International (now part of Millipore Corporation, Billerica, MA, USA), human TNFα was from ReliaTech (Wolfenbüttel, Germany), collagen was from Nycomed (now part of Takeda Pharmaceuticals International, Zürich, Switzerland), and anti-human tissue factor-FITC, anti-human P-selectin-RPE, and respective negative controls were from AbD Serotec (Oxford, UK). RNeasy Mini Kit, small interfering RNA (siRNA) against TNFR1 and TNFR2, and Effectene transfection kit were from Qiagen (Hilden, Germany), and predesigned glyceraldehyde-3-phosphate dehydrogenase (GAPDH), TNFR1, TNFR2, plasminogen activator inhibitor 1 (PAI-1), tissue factor, and thrombomudulin TaqMan primers were from Applied Biosystems (Foster City, CA, USA). All other chemicals were from Sigma-Aldrich (Munich, Germany).

### Animals

Animal experiments were performed in wild-type (WT) or TNFα receptor-deficient C57BL/6 mice. WT mice were purchased from Charles River (Sulzfeld, Germany). TNFR1^−/− ^and TNFR2^−/− ^mice were originally obtained from the Jackson Laboratory (Bar Harbor, ME, USA), and TNFR1^−/−^R2^−/− ^mice were subsequently generated by cross-breeding. Surgical procedures were performed under short-term anesthesia induced by a single intraperitoneal injection of midazolam 3 mg/kg (Ratiopharm, Ulm, Germany), fentanyl 0.03 mg/kg (CuraMED Pharma, Baden-Württemberg, Germany), and medetomidinhydrochloride 0.3 mg/kg (Pfizer, Berlin, Germany; produced by Orion Pharma, Espoo, Finland) diluted in 0.9% NaCl. After the experiments, the animals were killed by injection of an overdose (2 g/kg) of sodium pentobarbital (Merial, Hallbergmoos, Germany). All experiments were conducted in accordance with the German animal protection law and approved by the district government of Upper Bavaria (approval reference number AZ 55.2-1-54-2531-162-08). The investigation conforms to Directive 2010/63/EU of the European Parliament.

### Intravital microscopy in the dorsal skinfold chamber microcirculatory model

The dorsal skinfold chamber microcirculatory model was used in mice as described previously [23]. Animals with an intact microcirculation underwent carotid artery catheterization for application of drugs or injection of isolated platelets, respectively. Intravital fluorescence microscopy was performed by using a modified microscope (Zeiss Axiotech Vario; Carl Zeiss, Oberkochen, Germany). Images were recorded with a digital camera (AxioCam HSm; Carl Zeiss). For all *in vivo *experiments, TNFα was administered via carotid artery catheter at a dose of 0.4 µg/kg. This was calculated to match plasma levels of approximately 5 ng/mL, which caused effects *in vitro*.

### Intravital assessment of arteriolar thrombosis

Intravital thrombotic vessel occlusion time was assessed in arterioles of WT or TNFα receptor-deficient C57BL/6 mice in the dorsal skinfold chamber model. For induction of intra-arteriolar thrombosis, the ferric chloride superfusion method was used as described previously [23,24]. Before the experiments, blood vessel flow was digitally recorded and regular blood flow was confirmed for all analyzed arterioles. To visualize vessel lumina before vessel injury, 50 µL of a 5% fluorescein isothiocyanate-labeled dextran solution (FITC-dextran, molecular weight 150,000) was infused via the carotid catheter. Injury to the vascular wall was then performed by application of 30 µL of a ferric chloride solution (25 mmol/L) onto arterioles by using a standardized protocol, and movies were recorded until blood flow ceased.

### Mouse platelet isolation and staining for *in vivo *studies

Whole blood was drawn from anesthetized mice by cardiac puncture. To prevent blood from clotting, syringes contained 10% of sodium citrate. The citrated whole blood was spun at 130*g*, and the obtained platelet-rich plasma (PRP) was incubated with carboxyfluorescin (carboxyfluorescein diacetate succinimidyl ester 17 µmol/L; Bachem, Bubendorf, Switzerland) in the dark for 30 minutes. Labeled platelets were then spun at 340*g *and resuspended in a buffered calcium-free physiologic solution (138 mmol/L NaCl, 2.7 mmol/L KCl, 12 mmol/L NaHCO_3_, 0.4 mmol/L NaH_2_PO_4_, 1 mmol/L MgCl_2 _× 6 H_2_O, 5 mmol/L D-glucose, 5 mmol/L Hepes; pH 7.35). For centrifugation, iloprost (10 ng/mL; Schering, Berlin-Wedding, Germany) was added to prevent platelet activation. The ability of the isolated and stained platelets to aggregate was tested by platelet aggregometry.

### Intravital analysis of platelet-vessel wall interaction

For intravital studies of platelet interaction with the intact vessel wall, isolated and fluorescent-stained murine platelets from a donor animal were injected via a carotid artery catheter and observed in the dorsal skinfold chamber model. Movie sequences of 30 seconds in four- to six-vessel segments in each animal were recorded and analyzed by using AxioVision Software (Carl Zeiss). Vessels with abnormal flow were excluded from analysis. From the resulting length of the platelet trace in single images, velocities of single platelets were calculated by using the exposure time of each single picture. PVWI was expressed in frequency histograms consisting of all platelet velocities analyzed. Histograms were normalized to the maximum platelet speed within a vessel to exclude biasing influences of altered blood flow velocities between different arterioles. As a consequence, a rightward shift in platelet velocity distribution within a histogram expresses less PVWI, whereas a leftward shift signalizes increased PVWI at the arteriolar wall. Platelets with less than 5% of the velocity of the fastest platelets were defined as rolling platelets.

### Aggregation studies

Platelet aggregation was measured by using the turbidimetric method described by Born [25]. Human or murine PRP was obtained by centrifugation (130*g*) of whole citrated blood drawn from human cubital veins or by cardiac puncture in mice. ADP-, collagen-, or thrombin receptor-activating peptide (TRAP)-induced platelet aggregation was measured photometrically by using a two-channel aggregometer (ChronoLog 490-2D; Chrono-log Corporation, Havertown, PA, USA) under continuous stirring at 1,000 revolutions per minute at 37°C. Written consent was obtained from platelet donors. To assess the effects of endothelial-derived cytokines, 100 µL of supernatant of stimulated human microvascular endothelial cells (HMECs) was added to 300 µL of PRP and incubated for 90 minutes before agonist-induced aggregation was measured.

### Cell culture

Human umbilical vein endothelial cells (HUVECs) were isolated and cultured as described previously [26]. The procedure was approved by a university ethics review board. HMECs were provided by Ades and colleagues [27] and cultured in M199 media supplemented with 10% fetal calf serum, 10% endothelial growth media (PromoCell, Heidelberg, Germany), and 1% penicillin/streptomycin. The investigation conforms to the principles outlined in the Declaration of Helsinki.

### Measurement of endothelial superoxide (O_2 _^-^)

For superoxide measurements, the cytochrome c reduction method was used as previously described [28]. The O_2_^-^-dependent part of cytochrome c reduction was calculated from the difference in absorbance (550 nm) between samples incubated with or without superoxide dismutase.

### Endothelial surface molecule expression

HMECs or HUVECs were grown as described and incubated with sham or TNFα (5 ng/mL) as indicated. Cells were stained by using anti-p-selectin-RPE and anti-tissue factor-FITC or corresponding RPE- or FITC-labeled negative control. For measuring, a FACSCanto II flow cytometer (Becton, Dickinson and Company, Franklin Lakes, NJ, USA) was used. Data were analyzed by using FACSDiva software (Becton, Dickinson and Company).

### Real-time polymerase chain reaction in endothelial cells

HMECs were incubated with sham or TNFα for indicated time points. RNA isolation and real-time polymerase chain reaction (PCR) were performed as described previously [29]. Commercially available pre-developed TaqMan reagents were used for the human target genes thrombomodulin, PAI-1, tissue factor, TNFR1, and TNFR2, and GAPDH was used as a reference housekeeping gene. All measurements were performed in duplicates.

### Endothelial-dependent blood clotting

Cells were stimulated with TNFα as indicated and then lysed with 15 mM n-octyl-D-glycopyranosidase in 0.1 M imidazol buffer; 20 μL of cell lysate and 20 μL of 200 mmol/L CaCl_2 _for re-calcification were added to 300 μL of citrated (3.13% sodium citrate) human whole blood from healthy volunteers, and clotting time was measured by thrombelastometry (Rotem; Tem Innovations, Munich, Germany). Stimulation with human recombinant tissue factor was used as a positive control.

### Role of autocrine factors

To assess the role of TNFα-induced autocrine factors released by endothelial cells, cells were stimulated with TNFα 5 ng/mL for 3 hours. After removal of the TNFα-containing medium and a washing step, fresh medium without TNFα was added to the cells to allow secretion of prothrombotic factors. After 2 hours, this medium was used to stimulate untreated cells in which several parameters then were measured.

### Small interfering RNA knockdown

Endothelial cells were transfected with 50 nM siRNA against human TNFR1 and TNFR2 by using the magnetofection method in combination with the Effectene kit from Qiagen as previously described [30]. Transfected cells were left 48 hours to allow siRNA-mediated knockdown, which was confirmed by real-time PCR and Western blot, which was performed as described elsewhere [30].

### Statistical analysis

SigmaStat Software (SYSTAT, Chicago, IL, USA) was used to calculate statistical differences. Data were analyzed by using Student *t *test for normally distributed variables or the Mann-Whitney rank sum test, when normal distribution was not given. Data are expressed as mean ± standard error of the mean. Differences were considered significant when the error probability level was a *P *value of less than 0.05.

## Results

### Arteriolar thrombus formation *in vivo*

**Figure 1:**
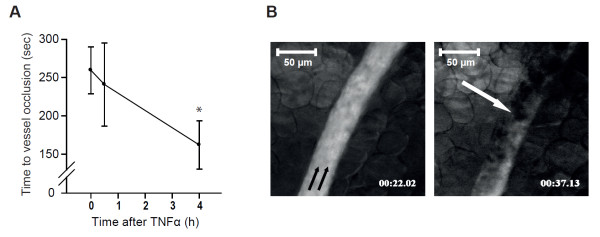
**Arteriolar thrombosis *in vivo***. **(a) **Time to thrombotic arteriolar vessel occlusion *in vivo*, as measured in the ferric chloride superfusion model in the dorsal skinfold chamber in mice, was significantly accelerated when animals were treated with tumor necrosis factor alpha (TNFα) (5 ng/mL) for 4 hours. **(b) **Thrombus formation after injury with ferric chloride. The arrow is pointing at a thrombus, which is spared by the fluorescein-labeled plasma and almost occluding the vessel. *Significantly different versus sham-treated animals at *P *<0.05 (n = 8 to 10).

To analyze the effect of TNFα on thrombus formation *in vivo*, we assessed the time to thrombotic arteriolar vessel occlusion following injury by ferric chloride superfusion to the vascular wall. The time to complete thrombotic vessel occlusion upon injury was significantly accelerated from 260 ± 31 seconds in control animals to 158 ± 31 seconds in animals treated with TNFα over a period of 4 hours prior to the experiment (n = 8, *P *<0.05 versus control animals). Vessel occlusion time was not significantly accelerated compared with control animals when TNFα was given only half an hour prior to the experiment (241 ± 54 seconds; n = 5; Figure 1a).

**Figure 2:**
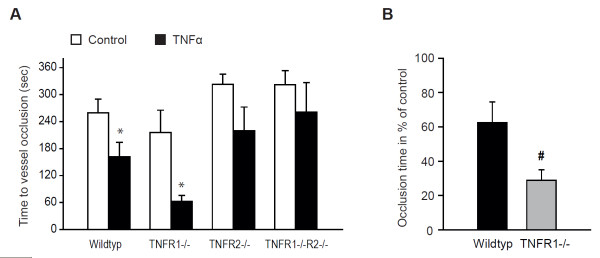
**Tumor necrosis factor alpha (TNFα)-induced arteriolar thrombosis *in vivo *is increased in TNFR1-deficient mice**. **(a) **Thrombotic arteriolar vessel occlusion *in vivo *was most accelerated with TNFα (5 ng/mL, 4 hours) in TNFR1-deficient mice. In mice lacking TNFR2 only or both TNF receptor subtypes, TNFα did not significantly affect arteriolar thrombosis. **(b) **In TNFR1^−/− ^mice, the relative acceleration of arteriolar thrombus formation by TNFα was significantly stronger compared with wild-type mice. *Significantly different versus respective sham-treated animals at *P *<0.05 (n = 5 to 10). ^#^Significantly different versus wild-type at *P *<0.05 (n = 5). TNFα, tumor necrosis factor alpha; TNFR1, tumor necrosis factor alpha receptor subtype 1; TNFR2, tumor necrosis factor alpha receptor subtype 2.

To investigate the contribution of signaling via the TNFα receptor subtypes, we measured time to thrombotic vessel occlusion in TNFR1^−/−^, TNFR2^−/−^, and TNFR1-/R2^−/− ^mice. The ability of TNFα to accelerate arteriolar thrombus formation *in vivo *observed in WT mice (Figure 1a) was further enhanced in TNFR1^−/− ^mice, in which the mean vessel occlusion time after treatment with TNFα was reduced to 62 ± 13 seconds (n = 5, *P *<0.05 versus sham-treated TNFR1^−/−^; Figure 2a), corresponding to a TNFα-dependent relative acceleration of thrombotic vessel occlusion time of 37% ± 12% in WT mice and of 71% ± 6% in TNFR1^−/− ^mice (n = 5 to 8; *P *<0.05 versus WT treated with TNFα; Figure 2b). In mice lacking TNFR2 or both TNFR1 and TNFR2, TNFα did not significantly accelerate thrombus formation *in vivo*. Without TNFα treatment, time to thrombotic vessel occlusion was not significantly affected in either of the TNFα receptor-deficient animals (Figure 2a).

### Platelet-endothelium interaction *in vivo*

Next, we assessed transient interaction of platelets to the vessel wall *in vivo *by intravital microscopy of vessels in the dorsal skinfold chamber. Following systemic TNFα treatment, interaction of injected labeled platelets with the endothelium was slightly enhanced as indicated by a leftward shift in platelet flow velocities and a decrease in the median platelet velocity from 4.0 mm/second in control animals (n = 3,294 platelets, 5 different animals) to 3.7 mm/second in TNFα-treated animals (n = 3,239 platelets, 5 different animals, *P *<0.05 versus control animals). The fraction of rolling platelets (platelets with less than 5% of the blood flow velocity) was only 0.1% ± 0.1% of all analyzed injected platelets in control animals and not significantly higher in TNFα-treated animals, in which it was 0.4% ± 0.6% (n = 5; data not shown).

**Figure 3:**
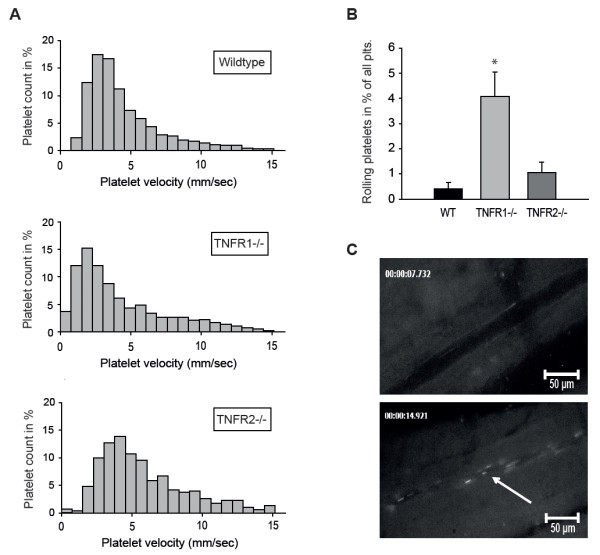
**Tumor necrosis factor alpha (TNFα) increases platelet-endothelium interaction and platelet rolling *in vivo *in TNFR1-deficient mice**. **(a) ***in vivo *platelet-endothelium interaction as analyzed by intravital microscopy in the dorsal skinfold chamber was greatly enhanced in TNFR1^−/− ^mice pretreated with TNFα (5 ng/mL, 4 hours) compared with wild-type (WT) or TNFR2^−/− ^mice treated likewise, as indicated by a leftward shift in platelet velocity distribution pattern in the frequency histogram of analyzed platelet velocities. **(b) **When rolling platelets (defined as platelets with velocity of less than 5% of maximum velocity) were quantified after TNFα treatment, the fraction related to all analyzed platelets was significantly higher in mice lacking TNFR1 compared with WT or TNFR2-deficient animals. **(c) **Digital records of carboxyfluorescein-labeled platelets in arterioles with increased platelet-endothelium interaction and rolling platelets (arrow). *Significantly different versus WT and TNFR2^−/− ^mice at *P *<0.01 (n = 3 to 5). plts, platelets; TNFR1, tumor necrosis factor alpha receptor subtype 1; TNFR2, tumor necrosis factor alpha receptor subtype 2.

In contrast, the leftward shift in platelet velocity pattern seen in WT mice after treatment with TNFα was much more prominent in TNFR1^−/− ^mice, indicating more interaction of platelets with the endothelium (Figure 3a). Moreover, TNFα increased the fraction of rolling platelets from 0.4% ± 0.3% in WT mice to 4.1% ± 1.0% in TNFR1^−/− ^mice (n = 5, *P *<0.01 versus WT; Figure 3b). In TNFα-treated TNFR2^−/− ^animals, the fraction of rolling platelets was not significantly different compared with WT mice (1.1% ± 0.4%). The maximum platelet velocities in the analyzed vessels as an approximate measure of flow velocity were not significantly different before and after TNFα treatment or between the genotypes.

### Platelet aggregation in platelet-rich plasma

**Figure 4:**
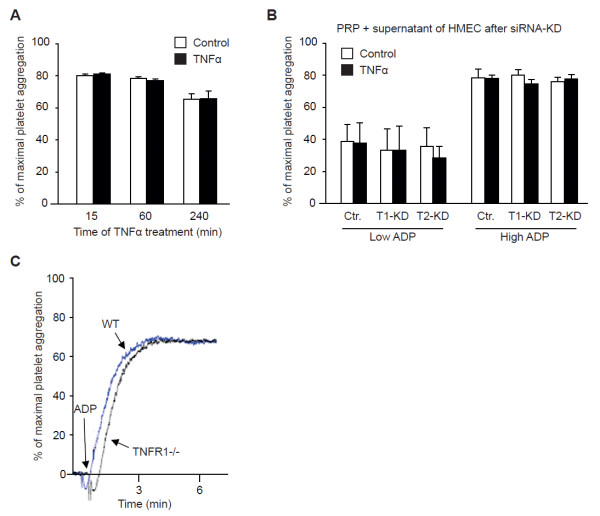
**Tumor necrosis factor alpha (TNFα**) **does not increase platelet aggregation in platelet-rich plasma (PRP) *in vitro***. **(a) **ADP-induced (5 μmol/L) platelet aggregation was not increased in human PRP stimulated with TNFα (5 ng/mL, 15 minutes to 4 hours) compared with non-stimulated PRP (n = 10). **(b) **Addition of supernatant from TNFα (5 ng/mL, 4 hours)-stimulated human microvascular endothelial cells (HMECs) after small interfering RNA (siRNA) knockdown of TNFR1 or TNFR2 to PRP for 90 minutes did not change platelet aggregation (n = 6). **(c) **No difference in platelet aggregation was observed after incubation with TNFα (5 ng/mL, 30 minutes) in PRP from TNFR1^−/− ^mice. The aggregation traces are representative of five independent experiments. Ctr., control; T1-KD, tumor necrosis factor alpha receptor subtype 1 knockdown; T2-KD, tumor necrosis factor alpha receptor subtype 2 knockdown; TNFR1^−/−^, tumor necrosis factor alpha receptor subtype 1 knockout; WT, wild-type.

To test for direct effects of TNFα on platelets, we assessed platelet aggregation *in vitro *in human PRP by using light transmission aggregometry (Born's method). Incubation of PRP with TNFα 5 ng/mL for different time intervals did not affect the ability of platelets to form aggregates upon stimulation with several commonly used platelet agonists - ADP (5 µmol/L), collagen (5 to 10 µg/mL), or TRAP (5 µmol/L) - either after a short time of incubation or after 4 hours (n = 8; results for ADP in Figure 4a). TNFα itself did not cause platelet aggregation either. Also, the addition of supernatant of TNFα-treated endothelial cells (with or without siRNA knockdown of TNFR1 or TNFR2) to PRP had no influence on platelet aggregation (n = 4; Figure 4b).

To check whether direct activation of platelets is responsible for prothrombotic TNFα effects in TNFR1^−/− ^mice, we performed platelet aggregation studies *ex vivo *in PRP from TNFR1^−/− ^mice. Like in human PRP, platelets from these animals showed no differences in the ability to aggregate with or without prior TNFα incubation (5 ng/mL) to different stimuli, including ADP and collagen, at different stimulation times (n = 5; Figure 4c).

### TNFα-induced prothrombotic changes in endothelial cells *in vitro*

**Figure 5:**
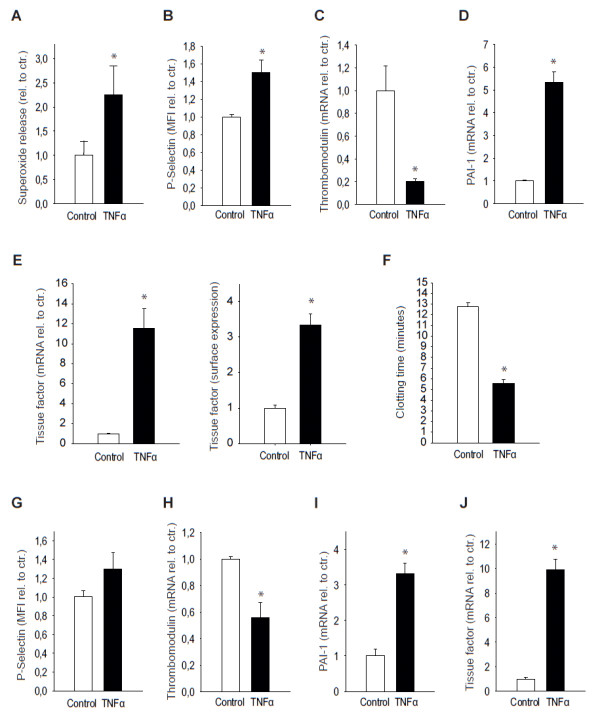
**Tumor necrosis factor alpha (TNFα) induces prothrombotic effects in endothelial cells *in vitro***. Treatment of endothelial cells with TNFα (5 ng/mL, 4 hours) led to increased superoxide release as assessed by cytochrome c reduction **(a) **and upregulation of P-selectin on the surface membrane **(b) **in human umbilical vein endothelial cells. Additionally, TNFα decreased thrombomodulin **(c) **and increased plasminogen activator inhibitor 1 (PAI-1) **(d) **as measured by real-time polymerase chain reaction in human microvascular endothelial cells (HMECs). **(e) **TNFα elevated tissue factor expression as well as protein on the cell surface membrane. **(f) **These changes resulted in significant acceleration of blood clotting time induced by lysates of TNFα-treated HMECs. **(g-j) **The supernatant of TNFα (5 ng/mL, 4 hours)-treated HMECs was used to stimulate untreated endothelial cells. This resulted in an upregulation of P-selectin after 30 minutes and prothrombotic changes in mRNA levels of thrombomodulin, PAI-1, and tissue factor which were qualitatively similar to, but less than those achieved by direct TNFα stimulation, indicating that autocrine factors secreted by TNFα-stimulated HMECs contribute to the prothrombotic phenotype. *Significantly different versus control at *P *<0.05 (n = 6 to 8). MFI, mean fluorescence intensity; rel. to ctr., relative to control.

To better define the relative contribution of the endothelium to TNFα-dependent prothrombotic effects, we analyzed pro- and anti-coagulant factors in primary human endothelial cells (HUVECs) and HMECs. We first measured the release of endothelial superoxide (O_2_^-^) upon TNFα stimulation in HUVECs by using the cytochrome c assay. The photometrically recorded superoxide dismutase-sensitive fraction of cytochrome c reduction, expressed as the difference in the absorbance, depicts the production of O_2_^- ^and was 2.2 ± 0.6-fold increased after stimulation with TNFα for half an hour in a dose of 5 ng/mL (n = 15, *P *<0.05 versus control; Figure 5a).

Next, we used flow cytometry to measure the adhesion molecule p-selectin, which is described to mediate platelet rolling on the endothelium [31]. Whereas p-selectin was barely detectable on the surface membrane of HUVECs either non-stimulated or stimulated with TNFα (5 ng/mL) for only 30 minutes, it was significantly upregulated after 4 hours, with an increase in the mean relative fluorescence of 50.4% ± 13.7% (*P *<0.05, n = 5; Figure 5b).

Thrombomodulin expression was found to be suppressed by 79.8% ± 2.4% upon treatment with TNFα 5 ng/mL for 4 hours (*P *<0.05, n = 8; Figure 5c). Furthermore, PAI-1, one of the most potent inhibitors of fibrinolysis, and tissue factor (CD142), which plays a crucial role in the activation of the extrinsic pathway of the coagulation cascade, were measured by real-time PCR in HMECs upon stimulation with TNFα for 30 minutes or 4 hours at 5 ng/mL. After 4 hours, mRNA levels of PAI-1 were more than fivefold higher compared with baseline (fold increase 5.3 ± 0.5; *P *<0.05, n = 16; Figure 5d), and both tissue factor expression and tissue factor protein on the surface membrane of HMECs were significantly upregulated after 4 hours of treatment with TNFα 5 ng/mL (11.6 ± 2.0-fold and 3.4 ± 0.3-fold increase versus baseline, respectively, *P *<0.05, n = 8 to 10; Figure 5e). Ultimately, in an endothelial-dependent clotting assay in whole blood, clotting time upon induction with HMEC lysates after stimulation with TNFα (5 ng/mL for 4 hours) was accelerated from 12.5 ± 0.3 minutes to 5.5 ± 0.4 minutes (*P *<0.05, n = 4; Figure 5f).

### Endothelium-derived mediators induce prothrombotic molecules

To test whether endothelial cells stimulated with TNFα secrete mediators which contribute to TNFα-dependent prothrombotic effects, HUVECs or HMECs were incubated for 30 minutes (for p-selectin) and 4 hours (for thrombomodulin, PAI-1, and tissue factor expression) with supernatants of cells previously stimulated with TNFα at 5 ng/mL for 3 hours. Whereas p-selectin was slightly but not significantly increased (n = 6; Figure 5g), expression levels of thrombodulin were reduced by 44% ± 12% (*P *<0.05, n = 4; Figure 5h), and mRNA levels of PAI-1 and tissue factor were significantly increased (3.3 ± 03-fold and 9.9 ± 0.8-fold increase versus baseline, respectively, *P *<0.05, n = 4; Figure 5i,j).

### Relative contribution of TNFR1 and TNFR2 in mediation of TNFα-dependent prothrombotic effects *in vitro*

**Figure 6:**
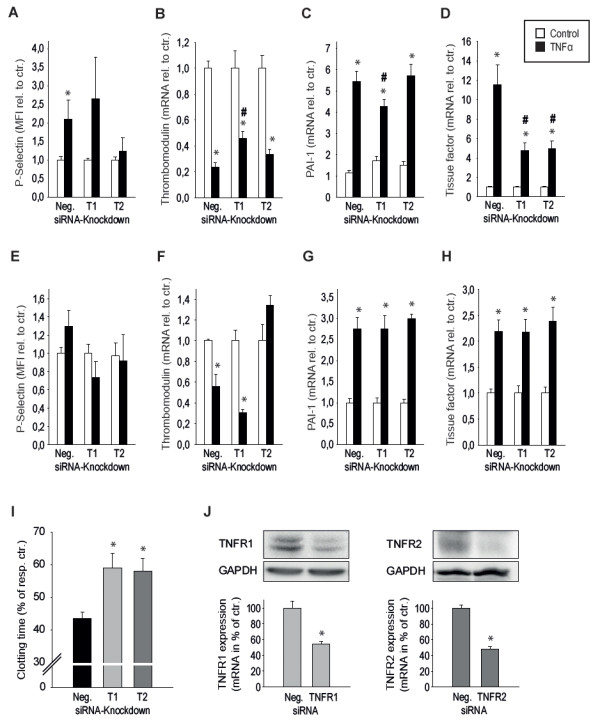
**Contribution of tumor necrosis factor (TNF) receptor subtypes to prothrombotic effects in endothelial cells *in vitro***. **(a) **TNFα (5 ng/mL, 4 hours)-induced upregulation of P-selectin is significantly reduced after TNFR2 knockdown. Whereas TNFα-dependent prothrombotic changes in thrombomodulin and plasminogen activator inhibitor 1 (PAI-1) expression were only reduced after TNFR1 knockdown **(b, c)**, single knockdown of TNFR1 and TNFR2 both significantly reduced TNFα-induced generation of tissue factor **(d) **and eventually acceleration of blood clotting induced by human microvascular endothelial cell (HMEC) lysates **(i)**. **(e-h) **The contribution of secreted autocrine factors (as assessed by stimulation with the supernatant of TNFα (5 ng/mL, 4 hours)-treated HMECs) to upregulation of P-selectin, PAI-1, and tissue factor was not changed after specific TNF receptor knockdown, while reduction of thrombomodulin was inhibited after knockdown of TNFR2. **(j) **Knockdown of TNFR1 (left) and TNFR2 (right) was achieved by small interfering RNA (siRNA) and magnetofection and was confirmed on mRNA as well as protein level as measured by real-time polymerase chain reaction and Western blot after 24 and 48 hours, respectively. *Significantly different versus respective control at *P *<0.05. ^#^Significantly different versus control-transfected cells treated with TNFα (n = 4 to 8 independent experiments). ctr., control; GAPDH, glyceraldehyde-3-phosphate dehydrogenase; MFI, mean fluorescence intensity; Neg., negative; rel. to ctr., relative to control; resp. ctr., respective control; TNFR1, tumor necrosis factor alpha receptor subtype 1; TNFR2, tumor necrosis factor alpha receptor subtype 2.

To define the relative contribution of the TNFR subtypes to induction of single genes relevant in thrombosis, knockdowns of TNFR1 and TNFR2 were performed in HMECs and HUVECs by using siRNA. As tested by real-time PCR and Western blot after 24 and 48 hours, respectively, for both receptors, a knockdown of about 55% could be achieved (Figure 6j).

Whereas upregulation of p-selectin was inhibited only by knockdown of TNFR2 (Figure 6a), TNFα-dependent changes in thrombomodulin and PAI-1 expression were significantly reduced by TNFR1 knockdown (Figure 6b,c). TNFα-induced upregulation of tissue factor was significantly reduced by knockdown of both TNFR subtypes (Figure 6d).

Thrombomodulin suppression induced by the supernatant of TNFα-stimulated HMECs was abolished after TNFR2 knockdown (n = 4; Figure 6f), and p-selectin, PAI-1, and tissue factor upregulations induced by soluble mediators released upon TNFα stimulation were not changed after knockdown of either of the TNF receptor subtypes (n = 4; Figure 6e,g,h). Knockdown of both TNFR1 and TNFR2 significantly inhibited endothelial-dependent TNFα-induced acceleration of blood clotting (*P *<0.05, n = 4; Figure 6i).

## Discussion

This study provides evidence of TNFα significantly accelerating ferric chloride-induced thrombus formation in an *in vivo *mouse model for arteriolar thrombosis. Its prothrombotic effects *in vivo *require the endothelial expression of TNFR2 and are more pronounced in the absence of TNFR1. *in vitro *studies indicate endothelial mechanisms to be responsible for prothrombotic TNFα effects, including an enhanced endothelial production of reactive oxygen species; upregulation of p-selectin, tissue factor, and PAI-1 expression; and suppression of thrombomodulin. TNFα results in the release of soluble mediators by endothelial cells which induce prothrombotic and suppress anticoagulant genes similar to direct TNFα-effects, but to a lesser extent. The early occlusion of capillary vessels might well be relevant in autoimmune disorders, as both endothelial cell activation and an enhanced influx of inflammatory cells are able to promote disease manifestations, and, in fact, microvessel changes have been a long-standing diagnostic tool in nailfold capillary microscopy and are well defined in lupus nephritis.

Our findings are consistent with the previously characterized direct effects of TNFα on endothelial cells, such as the induction of pro-inflammatory cytokines, fibrin deposition, and an increase in permeability [15-17,32]. In contrast, *ex vivo *platelet aggregation in our study was not influenced either by TNFα itself or by soluble mediators released by endothelial cells upon stimulation with TNFα. Of note, a weak direct activation of platelets by TNFα has been described [33,34]; indeed, from our *in vivo *results, we cannot exclude a direct effect of TNFα on platelets. In regard to our *in vivo *studies, we do explicitly consider our results not to be conflicting with the prior experimental evidence for antithrombotic activity of TNFα in a short time stimulation protocol [35]. In our model of arteriolar thrombosis, we observed a significantly accelerated time to thrombotic vessel occlusion upon injury 4 hours after systemic treatment with TNFα, and this is consistent with other studies of thrombosis in an inflammatory milieu [36]; after 30 minutes, neither pro- nor anti-thrombotic effects were seen, and this could be explained by differences in the injury model as well as in the vascular bed.

In this work, we compared arteriolar thrombus formation and platelet-endothelium interaction in mice deficient in TNFR1 or TNFR2 and animals lacking both TNFα receptor subtypes. Just as the site- and time-dependent context of its release, the complex interplay between TNFα receptor subtypes is pivotal in defining the actual effect of TNFα [3]. In addition, though sharing the same signaling pathways, responses of immune cells differ from those of non-immune cells, which ultimately cause organ-specific pathology in chronic inflammation [37] and hence represent equally important targets in the therapy of autoimmune diseases. Not only is the expression of TNFR2 highly regulated, but the stimulation of TNFR2 encompasses the induction of endogenous TNFα, and reverse signaling through tmTNFα that influences expression of cytokines and adhesion molecules in the tmTNFα-bearing cell has been described [38]. In regard to vascular biology in particular, TNFR2 seems to be crucial for both TNFα-induced atherosclerotic lesions in mouse models for atherosclerosis [39-41] and leukocyte interaction with the endothelium [42]. Our observations indicate that TNFR2 is required to mediate the prothrombotic effects of TNFα *in vivo*, whereas TNFR1 partly compensates these effects, since the prothrombotic effect of TNFα is potentiated in TNFR1 knockout mice when compared with WT animals. However, activation of TNFR1 does not seem to have antithrombotic activity itself but is merely able to attenuate the prothrombotic effects mediated by activation of TNFR2; indeed, TNFR2 knockout mice do not show a significantly longer vessel occlusion time than mice either not pretreated with TNFα or lacking both TNFR subtypes.

As platelets from TNFR1-deficient mice *ex vivo *do not aggregate more than WT platelets, TNFR2-mediated prothrombotic effects *in vivo *are likely not to rely on direct effects on platelets. Hence, we investigated TNFα-dependent changes in the expression of prothrombotic molecules in endothelial cells *in vitro *after siRNA knockdown of TNFR1 or TNFR2. According to the conclusion from the *in vivo *experiments, in which prothrombotic effects required TNFR2 and were more pronounced in the absence of TNFR1, mediators of a procoagulant milieu were expected to be less inducible by TNFα in the TNFR2 knockdown cells. This could be confirmed for p-selectin; however, TNFα-dependent induction of PAI-1 and suppression of thrombomodulin seemingly depended predominantly on TNFR1, while both TNFR subtypes were required for the upregulation of tissue factor and endothelial-dependent clotting. Interestingly, normal expression of TNFR2 was also necessary for the secretion of soluble mediators causing the suppression of thrombomodulin. Discrepancies to *in vivo *experiments can be explained by the incomplete receptor knockdown *in vitro*, which implicates a persistent interplay between the receptor subtypes. In addition, the analysis of single target genes in the endothelium certainly does not entirely reflect the complex setting of inflammation and coagulation *in vivo*.

With respect to clinical implications, the relevance of experimental results to most clinical conditions in humans is not clear as the majority of studies have been done in genetically deficient mice. However, anticytokine approaches with soluble receptor antagonists and monoclonal antibodies have become a mainstay in therapy of RA and other chronic inflammatory diseases. So far, there is no evidence of an increase in thrombotic events in patients treated with TNFα antagonists [43]. In this context, the finding that prothrombotic TNFα effects are mediated by TNFR2 is intriguing as deterioration of microcirculation in inflammation could well be relevant with respect to drug delivery, particularly prone to be compromised at the sites defined as therapeutic targets. Maintenance or reconstitution of an unhampered blood flow should be attained particularly well by a more selective therapeutic approach consisting of a TNFR2-specific antagonist. The implementation of TNFR2-specific antagonists has already been advocated because the highly regulated receptor subtype TNFR2 mediates tissue damage in chronic inflammation. A selective blockade of TNFR2 would also allow downregulation of excessive TNFα signaling in uncontrolled inflammatory processes while leaving unaffected beneficial TNFα effects. In addition, amplification of TNFR1 responses by ligand passing and reverse signaling through tmTNFα would both be abolished by antagonizing TNFR2. Still, with regard to the improvement of therapeutic strategies, we consider the observation of soluble, endothelium-derived mediators to be very important as they presumably allow a local amplification of prothrombotic responses in inflammation.

## Conclusions

We hereby provide evidence for prothrombotic effects of the inflammatory cytokine TNFα. In our *in vivo *model of arteriolar thrombosis, both prothrombotic effects and increased platelet rolling are mediated through endothelial TNFR subtype 2 and partly compensated for by TNFR subtype 1. *In vitro*, both TNFα itself and a TNFα-induced release of soluble mediators led to an induction of prothrombotic and a suppression of anticoagulant molecules in human endothelial cells, resulting in an accelerated clotting time in endothelial cell-based assays. Assuming that the occlusion of capillary vessels in autoimmune disorders is relevant well beyond mere thrombosis, we argue in favor of a more selective therapeutic approach in anticytokine therapy based on TNFR2-specific antagonists.

## Abbreviations

FITC: fluorescein isothiocyanate; GAPDH: glyceraldehyde-3-phosphate dehydrogenase; HMEC: human microvascular endothelial cell; HUVEC: human umbilical vein endothelial cell; NF-κB: nuclear factor-kappa-B; PAI-1: plasminogen activator inhibitor 1; PCR: polymerase chain reaction; PRP: platelet-rich plasma; PVWI: platelet-vessel wall interaction; RA: rheumatoid arthritis; RPE: R-phycoerythrin; siRNA: small interfering RNA; tmTNFα: transmembrane tumor necrosis factor alpha; TNFα: tumor necrosis factor alpha; TNFR1: tumor necrosis factor alpha receptor subtype 1; TNFR2: tumor necrosis factor alpha receptor subtype 2; TRAP: thrombin receptor-activating protein; WT: wild-type.

## Competing interests

The authors declare that they have no competing interests.

## Authors' contributions

JP designed the study, performed the *in vivo *experiments, and wrote the manuscript. MM and MW wrote the manuscript and analyzed and interpreted the data. TC and YS performed *in vivo *experiments. HM, AR, and MN did the *in vitro *experiments. VV provided the knockout mice and revised the manuscript. FK closely supervised the study and did the final revision for intellectual content. All authors read and approved the final manuscript.
